# Facial asymmetry correction: From conventional orthognathic treatment to surgery-first approach

**DOI:** 10.15171/joddd.2019.047

**Published:** 2019

**Authors:** Tahereh Hosseinzadeh Nik, Elaheh Gholamrezaei, Mohammad Ali Keshvad

**Affiliations:** ^1^Department of Orthodontics, School of Dentistry, Tehran University of Medical Sciences, Tehran, Iran

**Keywords:** Surgery first approach, facial asymmetry, skeletal deviation, orthognathic surgery

## Abstract

The surgery-first approach (SFA), which proceeds without presurgical orthodontic treatment, is assumed to shorten the treatment course because the direction of post-surgical orthodontic tooth movement conforms to the normal muscular forces. Moreover, the regional acceleratory phenomenon (RAP), evoked by surgery, helps in tooth alignment and compensation in a faster way. Although SFA has definite advantages, especially in class III individuals, there is a lack of data about its indications in patients with facial asymmetry. In this article, we reviewed recently published articles on the treatment of asymmetric patients using the SFA. Different aspects, including the three-dimensional assessment of stability in different planes, approaches for fabrication of a surgical splint, predictability of the results, skills needed for bimaxillary surgery, indications as the treatment of choice for condylar hyperplasia, and combination with distraction osteogenesis in candidates with severe asymmetries were found to be the main topics discussed for patients presenting with facial asymmetry

## Introduction

Orthognathic surgery is a common oral surgical procedure indicated for patients with severe skeletal discrepancy and unaesthetic profile, who require a treatment procedure more invasive than orthodontic tooth movement alone. Routinely, this process includes leveling and alignment of the teeth, dental decompensation, and arch coordination, which takes 12‒24 months, depending on the severity of malocclusion, and should be performed preoperatively.^1^ During this period, however, progressive deterioration of facial esthetics and masticatory function occurs due to dental decompensation, and patients often complain of various levels of pain.^2^ Also, changing the dentoalveolar condition during the decompensation period is difficult due to tight occlusion and soft tissue imbalance.^3^ On the other hand, it is believed that surgeons would have limitations in correcting skeletal deformity without appropriate presurgical orthodontic treatment due to improper positioning of the teeth. Thus, all the therapeutic goals included in the treatment plan may not be achieved without appropriate presurgical orthodontic treatment.^4,5^

The surgery-first approach (SFA) is a novel technique, which has recently gained increasing popularity among orthodontists and oral and maxillofacial surgeons and does not require presurgical orthodontics. It appears to shorten the course of treatment by 1 to 1.5 years.^6^ It is believed that the direction of orthodontic tooth movement after surgery is coordinated with muscular forces that accelerate tooth alignment and dental decompensation.^7^ In addition, the regional acceleratory phenomenon (RAP) is activated by the orthognathic surgery as part of the wound healing process and can play an important role in shortening of the treatment course.^8,9^

**Figure 1:**
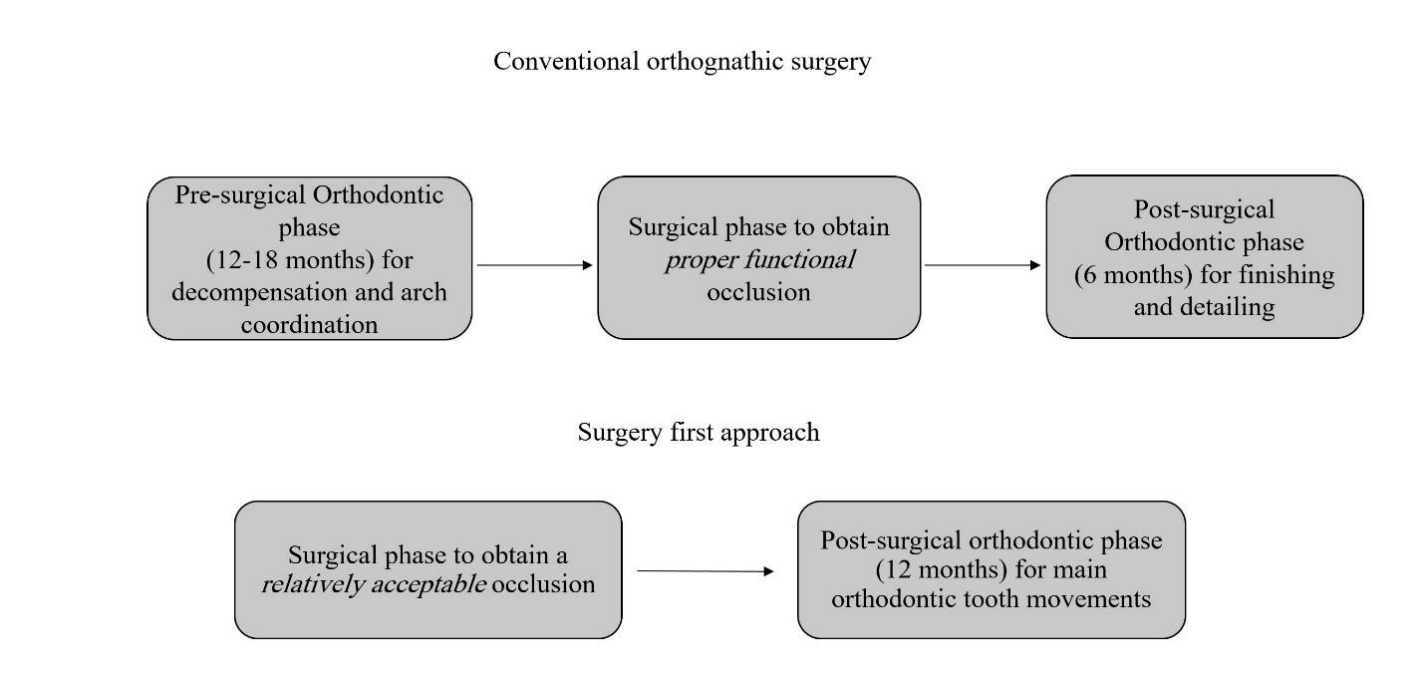
Comparison of conventional orthognathic surgery with the surgery-first approach (SFA).

Evidence shows that the serum level of alkaline phosphatase, which is a key enzyme in bone formation, increases as part of the RAP process four weeks after surgery, and its level remains high for up to four months postoperatively.^10^ The absence of a tight occlusion at the time of orthognathic surgery is a double-blade sword. It can enhance the movement of teeth that are no longer locked in the occlusion and decrease the possibility of iatrogenic complications of treatment, such as root resorption. However, with regard to stability, it is believed that the absence of maximum inter-digitation immediately after surgery can lead to a high risk for relapse in the future.^11^ A comparison of these two approaches is illustrated in Figure 1.

Similar to all the procedures, the SFA has some shortcomings as well, such as difficult prediction of the final occlusion, inaccuracy in treatment planning of patients requiring tooth extraction and long process of treatment planning, which requires several consultation sessions between the surgeon and orthodontist.^12^ Since the introduction of this approach, many clinical and review studies have evaluated its efficacy, especially in class III patients.^13,14^ However, difficult presurgical orthodontic treatment in patients with facial asymmetry and difficult prediction of outcome and its stability, as well as its high popularity among surgical patients, prompted us to carry out a comprehensive review regarding SFA in patients with facial asymmetry due to trauma, uncontrolled growth as in condylar hyperplasia or other factors. This study aimed to review and compare conventional orthognathic surgery and SFA in patients with facial asymmetry due to different reasons.

## Methods

As the first step in this literature review, we developed a specific research question regarding PICO format, as illustrated in Table 1. An electronic search was performed in PubMed, Scopus, MEDLINE, EMBASE, Cochrane Library, and Google Scholar databases. Hand searching of the reference lists of the included studies was also performed. The searched keywords included “facial/frontal asymmetry AND surgery first [approach]”, “skeletal deviation AND surgery first [approach]” and “condylar hyperplasia/hypertrophy/fracture AND surgery first [approach]”. The inclusion criteria for the selection were: 1) English articles; 2) publishing date from 2000 to 2019; 3) human subjects with skeletal deviation; and 4) no history of presurgical orthodontic treatment. The exclusion criteria were: 1) Articles discussing the surgery-first approach in symmetric patients; 2) any orthognathic procedure except the surgery-first approach; and 3) a history of presurgical orthodontic treatment. No limitations regarding the type of articles were defined.

**Table 1:** PICO format

Population	Patients with facial asymmetry
Intervention	Surgery first approach
Comparison	Conventional orthognathic surgery; if possible
Outcome	Esthetic, stability and functional outcomes

Two orthodontics residents and an orthodontic professor independently reviewed the titles and abstracts. The full texts of the articles that seemed to meet the inclusion criteria were reviewed thoroughly, and data collection forms were used for data abstraction. The differences in opinions were resolved by consensus, and the data were excluded if an agreement could not be reached. The last search was performed in May 2019.

## Results

**Figure 2:**
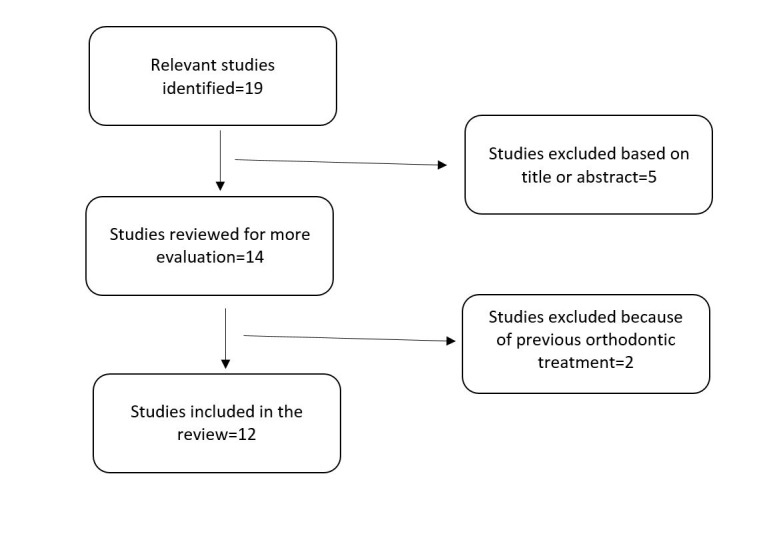
Flow diagram.

According to Figure 2, of 19 relevant studies, 12 articles met our inclusion criteria, six of which were case reports, and six were retrospective. The main topics discussed in the articles included SFA stability and its functional and esthetic consequences (7 studies), changes in condylar position after surgery (2 studies), indication in patients with condylar hyperplasia (2 studies) or those with congenital disorders (1 study). Three-dimensional diagnosis and treatment planning and custom-made modifications in this process for patients were emphasized in 3 of these articles. The largest sample size was 65. Five studies had a postoperative follow-up of ≥1 year. Two studies had conventional surgery as the control groups, and one study compared SFA in symmetric and asymmetric patients. Except for one case report, which used SFA in an asymmetric patient with class II malocclusion and another one about congenital disorders, the samples in the ten remaining studies showed class III skeletal pattern in addition to facial deviation. Almost none of the studies described any other reasons than the reduction of treatment time, for choosing SFA. The extracted data included the study design, sample size, type of malocclusion, interventions, and findings, which are presented in Table 2. The details are discussed below.

**Table 2:** Summary of the findings

**Ref/year**	**Article type**	**Aim**	**Sample**	**Intervention**	**Finding**
**Carlo Villegas** ^15^ **2010**	Case report	Evaluation of esthetics and stability of SFA	1 sample with ClIII malocclusion and mandibular deviation	Surgery first-asymmetrical single-jaw surgery. miniplates were used for orthodontic correction after surgery	Good stability and esthetic results, no signs of relapse after 6-month follow-up
**Jiyin Li** ^16^ **2017**	Retrospective study	evaluate changes in condylar position after SFA	BSSRO-only group: 12 patients BSSRO with Le Fort I osteotomy group: 6 patients. All were asymmetric patients.	Scans were obtained before surgery (T0), 3 days postoperatively (T1), and 6 months postoperatively (T2).	Same bodily shift and rotational changes of condyle in 2 surgical treatment plans. Deviated side showed more displacement than non-deviated side.
**Jinyuan Guo** ^17^ **2018**	Retrospective study	To evaluate corrective outcome and transverse stability in SFA using 3-dimensional analysis	17 symmetric and 12 asymmetric patients.	CT scan comparison at 3 time points assessing facial asymmetry indices including maxillary height, ramal length, frontal and lateral ramal inclination, mandibular body length and height	reduction of discrepancies between deviated and non-deviated sides for all indices after surgery which was stable during follow up
**Min-Hee Oh** ^18^ **2017**	Retrospective study	To compare the condylar displacement between the conventional approach versus surgery-first approach	38 patients divided into 2 groups: 18 conventional group and 20 SFA group	CBCT taken before and 1 month after surgery. The condylar displacement was evaluated in the 3 Axes.	Condylar displacement showed no statistically significant differences between the two groups except for deviated side of conventional group
**Diego Fernando López** ^19^ **2017**	Case report	SFA in a Class III asymmetric patient with condylar hyperplasia	a 15-year-old female was diagnosed with Unilateral Condylar Hyperplasia by mean of SPECT and histological study	A high condylectomy and SFA 1 month later	Excellent facial and occlusal outcomes were obtained and after 24 months in retention the results remained stable.
**Nandacumr Janakiraman** ^20^ **2015**	Case report	The use of 3D digital technology and the SFA in a Class III patient with facial asymmetry caused by unilateral condylar hyperplasia	A 23 years old woman with facial asymmetry caused by unilateral condylar hyperplasia	3D computer aided surgical and orthodontic planning including fabrication of surgical splints using the CAD/CAM technique, and prediction of final orthodontic occlusion using robotically assisted customized archwires.	Excellent esthetic and occlusal outcomes were obtained in a short period of 5.5 months.
**Flavio Uribe** ^21^ **2013**	Case report	SFA by means of 3D computer-aided surgical planning based on CBCT scan procedure	Two patients with skeletal asymmetry in addition to ClII and ClIII malocclusion	3D CBCT-based treatment planning for the surgical correction of facial asymmetry in conjunction with the SFA.	Good esthetic and occlusal outcomes with a short total treatment time. Movements preformed during operation were similar to predicted 3D surgical movements
**Yorikatsu Watanabe** ^22^ **2019**	Case report	Combination of SFA and DO in hemifacial microsomia 5 adult patients with severe facial asymmetry	5 adult patients with severe facial asymmetry	Ideal maxillary positioning with SFA and mandibular traction via internal device toward maxilla –no comparison group	Successful treatment with good esthetics and without any complication.
**Han-Sol Song** ^23^ **2017**	Retrospective study	To evaluate transverse dental changes in class III and asymmetric patients undergone SFA and conventional orthognathic surgery	16 patients with conventional surgery and 13 patients s with SFA	CT scan evaluation before, immediately after and 1 year after surgical treatment	No significant dental and skeletal differences after 1 year between two groups
**Hyeon-Shik Hwang** ^24^ **2017**	Case report	describes a guideline for SFA in patients with facial asymmetry	A 19-year-old woman presented with severe mandibular prognathism and facial asymmetry.	2-jaw surgery including differential mandibular setback by SFA. Brackets were bonded after surgical process. Comparison of changes were made by CBCT and cephalometric tracings.	Patient's facial appearance improved significantly and a stable surgical outcome was obtained. Condyle on the deviated side moved inferiorly after surgery
**Yu-Fang Liao** ^25^ **2018**	Retrospective study	Evaluate the outcome of bimaxillary surgery for asymmetric skeletal Class III deformity using SFA	65patients with asymmetric skeletal Class III deformity.	Comparison of the values concerning symmetry from pre and post-surgical photographs plus filling questionnaire about patients’ satisfaction.	Significant improvement in the facial midline, facial contour, and overall facial symmetry. Questionnaires showed that patient satisfaction was high.
**HB Yu** ^26^ **2015**	Retrospective study	Report the experiences about the SFA for skeletal malocclusion	50 patients with skeletal malocclusions (11 bimaxillary protrusion, 27 skeletal class III malocclusions, and 12 facial asymmetry)	Surgeries included Le Fort I maxillary osteotomy, BSSO, subapical osteotomy, and genioplasty. Postoperative orthodontic treatment was started after 2 weeks.	Good facial profiles were achieved. With the advantages of earlier improvements in patient facial aesthetics and dental function, SFA is regarded as an ideal method

## Discussion

Most researchers believe that >4 mm of deviation from the mid-sagittal plane or facial midline can be detected as asymmetry by the laypeople.^27-30^ As explained earlier, the prevalence of asymmetry depends on the method of measurement and analysis. Thus, variable prevalence rates have been reported in the literature, ranging from 11% in a 5-year study^31^ to 55.2%, 27.2% and 17.6% for the three levels of mandibular asymmetry namely mild, moderate and severe asymmetry, respectively, in a recent study by Thiesen,^32^ using CBCT scans of 1,178 patients. Trauma, condylar fracture, fetal anomalies, syndromes, and pathologies such as rheumatoid arthritis can lead to facial asymmetry; however, the majority of orthodontists are uncertain about the reason and etiology of most asymmetries.^28^

The need for presurgical orthodontics is minimized or excluded in the SFA.^11,33^ Considering the existing concerns in this respect, a recent systematic review revealed that in both jaws, the SFA and the conventional orthognathic surgery had no significant difference concerning post-surgical stability and the range of possible surgical movements (in terms of magnitude). Although they could not assess the quality of life of patients in their meta-analysis, they reported that the quality of life was more favorable preoperatively in the SFA group because facial attractiveness was not compromised by preoperative orthodontic treatment. They suggested that orthodontists should inform their patients of the longer course of postoperative orthodontic treatment, which is a part of the SFA.^34^ The same results were reported in a systematic review by Soverina et al, who indicated similar stability in both methods but suggested to assess the stability of the results a couple of months after debonding and not a few months after surgery.^35^ Despite the large number of articles available on the SFA, only a few of them have evaluated patients with asymmetry and application of SFA for such patients. In order to carry out a comprehensive review regarding SFA in patients with asymmetry, we classified the findings of the 12 reviewed articles in 3 categories as follows.

### 1. General guidelines and information regarding the short-term and long-term stability of treatment outcomes

Liao et al^25^ defined facial asymmetry as ≥4 mm of deviation of menton from the facial midline. They presented specific guidelines for the repositioning of the jaws during surgery. According to these guidelines, in the vertical plane, due to late proclination of mandibular incisors and extrusion of mandibular posterior teeth during leveling and alignment of the arch, the models should be positioned in a relationship with positive overjet and posterior open bite within the limits of orthodontic movements (<10 mm). Due to the absence of decompensation process during the SFA, molar relationship, rather than incisor and canine relationships, determines the anteroposterior relation of the jaws because incisors are not in their correct position relative to the basal bone at the time of surgery. These findings are similar to the previous results reported by Yang Zhou et al.^36^ In the transverse plane, due to the facial asymmetry, the jaw midline during surgery is adjusted according to the facial midline, and this is the most important step in this treatment plan. They suggested occlusal adjustments and bite opening as the two main strategies to achieve the most stable occlusion during surgery and eliminate the possible interferences of the teeth. Sixty-five patients were followed up for one year after debonding, and their facial symmetry was evaluated by angular measurements and assessment of the deviations of the upper face, midface, and lower face contours on patient photographs. For a subjective analysis, the patients were requested to fill out a questionnaire regarding their satisfaction with treatment. Except for the contour of the midface, all the variables, such as the inter-commissural line, chin deviation, and midface deviation, significantly improved after the SFA. They concluded that significant changes in the upper face contour indicate the effect of movement of the proximal segment of the mandible during bilateral sagittal split osteotomy (BSSO) on the improvement of ramal symmetry. Moreover, 46 patients (71%) also underwent genioplasty along with orthognathic surgery, which could have also improved the symmetry of this part of the face in addition to the effect of changes in the distal segment of the mandible. The questionnaires revealed an acceptable level of patient satisfaction with their facial appearance, but some interesting results were also achieved. The patient satisfaction was the highest with the tooth and chin position and the lowest with the nose position postoperatively. The authors believed that postoperative nasal changes, such as the widening of the alar base, could be responsible for patient dissatisfaction in this respect. Eventually, they reported that the mean improvement reported by patients was significantly higher than the improvement observed in photographs. However, it should be noted that they did not follow the patients for a long time.

In an attempt to certify the findings regarding the SFA, Guo et al^17^ assessed the transverse stability of the SFA in 29 Class III patients by three-dimensional analysis. The CT images were compared at three time intervals of before, immediately after, and six months after surgery. They concluded that in the asymmetric group (n=12 patients), the difference in all the parameters between the symmetric and asymmetric sides decreased postoperatively. These parameters included the mandibular and maxillary body height, mandibular body and ramus length, and ramus inclination. These reductions were still stable after six months. A comparison of patients with symmetrical and asymmetrical face after surgery revealed no difference in these parameters between the two groups (indicating the ideal correction of asymmetry) except for the difference in mandibular body and ramus length in the asymmetric group, which showed a difference between the two sides at six months. However, this difference was not clinically significant. An interesting finding was that there was no significant difference in the overall treatment time between the symmetric and asymmetric groups. The SFA in their study shortened the course of treatment by 10 months. Considering the fact that the stability of SFA is not very clear, especially in patients with asymmetry,^37^ they stated that achieving a stable occlusion with the SFA is difficult due to the absence of decompensation, and this can possibly have a destructive effect on stability, as stated earlier by Baek.^38^ The use of a surgical splint in such conditions is a necessity as mentioned by Nagasaka.^39^ However, some other preventive and adjunctive methods have been suggested in the literature to stabilize the results. In a case report, Villegas et al^15^ used four mini-implants in the infra-zygomatic region and the external oblique ridge in both jaws to enhance post-surgical tooth movement. Due to the stable occlusion of the patient, they did not use a splint. However, at the end of treatment, they retained the mini-implants at the site for six months to use them immediately in case of relapse. Accordingly, Hwang et al,^24^ in a case report, provided post-surgical stability by using elastics and mini-screws at the site of premolar‒canine within the first five weeks postoperatively.

Guo et al^17^ also mentioned that frontal ramal inclination in the proximal segment had a significantly greater effect on patients’ perception of symmetry compared to the lateral inclination of the ramus. Thus, managing the perioperative rotation of the proximal segment at the time of surgical fixation is an important factor involved in postoperative relapse and can compromise the esthetic results in patients with mandibular asymmetry. Due to the elongation of pterygomasseteric sling, the surgeons should try to preserve the corrections made in the frontal inclination of the ramus proximal segment for better stabilization.^40,41^ Guo et al^17^ believed that semi-rigid fixation with mono-cortical miniplates and screws is suitable for this purpose and can stabilize the proximal segment after asymmetrical BSSO in patients suffering from deviation. Their findings revealed that the differences in frontal inclination of the ramus at the two sides of the face of asymmetric patients increased by <1º in the post-surgical phase, which was not clinically significant.

The majority of studies have evaluated post-surgical changes in the sagittal plane, while Song et al^23^ evaluated transverse (buccolingual) changes in the tooth axis on CBCT images of 29 class III patients with asymmetry after SFA and conventional orthognathic surgery.They performed vertical osteotomy of the ramus (VSO) instead of BSSO. The conventional group underwent one year of orthodontic treatment prior to surgery. However, CBCT measurements revealed no significant differences in the buccolingual inclination of maxillary and mandibular molars between the two groups preoperatively. The maxillary first molar on the deviation side and the mandibular first molar on the side without deviation had a lingual inclination on the CBCT scan taken immediately after surgery, which was different from the buccal inclination of mandibular first molar on the deviated side and maxillary first molar on the side without deviation. The difference in axial inclination was not significant between the two groups. Skeletal parameters such as the difference in the inclination and length of the ramus showed improvements on both sides postoperatively. This finding was consistent with the results reported by Guo et al.^17^ However, one year after surgery, the molar and canine inclination in the conventional group remained more stable. The researchers concluded that compared to the presurgical state, no significant difference was noted in the clinical skeletal and dental variables between the two approaches and stated that physiological adaptation decreased the speed of tooth movement before the surgical procedure, while the position and inclination of the teeth after surgery in the SFA group changed in an accelerated fashion, and decompensation occurred more easily. They also added that uncertainty in the prediction of axial changes of the posterior teeth after SFA should prompt the surgeons to use a splint to achieve higher and more stable occlusal contacts during jaw repositioning.

### 2. Changes in the condylar position after surgery

Three-dimensional repositioning of bone segments in patients with asymmetry is of particular importance.^42^ This can lead to displacement and errors in the repositioning of the condyles, especially during asymmetrical set-back surgery. This factor plays a critical role in surgical relapse.^43-46^ Oh et al^18^ evaluated linear displacement of the condyle in 38 patients after mandibular set-back surgery. Patients with asymmetry underwent conventional orthognathic surgery or the SFA, and their lateral and posteroanterior cephalograms obtained from their CBCT scans were evaluated. They assessed mediolateral, superoinferior, and anteroposterior dimensions.However, they did not assess angular changes. Significant condylar displacement was noted in both groups after surgery, with no difference between them. They noticed downward and backward rotation of the condyle in both the deviated and non-deviated sides in the SFA group, whereas only the downward rotation of the condyle was reported on the deviated side in the conventional group. The non-deviated side in this group showed downward and backward rotation of the condyle, similar to the other groups. No significant association was noted between asymmetrical set-back and condylar displacement. Wang et al^47^ evaluated condylar displacement in class III patients after the SFA, and similar to Oh et al,^18^ found no significant difference between the two surgical approaches; however, their patients did not have asymmetry. Oh et al^18^ also emphasized that a CT scan taken in the upright position is more similar to the natural head position and is more suitable for the assessment of the position of the condyles.^48^ However, they did not have long-term data, and the CT scans had been taken at different time intervals (three weeks after surgery in the conventional group and four weeks after surgery in the SFA group). Nonetheless, their findings were consistent with those of other studies on this topic.^41,49^ Previous studies have mentioned the technique of surgery (VSO/BSSO), the method of osteotomy, the technique of fixation, alignment of bone segments, method of repositioning of the condyles, the rotational movements of this segment, and presence of asymmetry as the key factors in the condylar displacement and its stability.^42,46,50^ Although some studies recommend the use of a condylar repositioning tool for higher accuracy, the positive efficacy of such tools has not yet been confirmed.^51^

In order to assess the effect of maxillary surgery on the condylar position, Li et al^16^ classified patients with skeletal class III malocclusion and facial asymmetry based on the presence/absence of LeFort I osteotomy in their surgical plan. The CT scans were obtained at three time intervals, i.e., before surgery (T0), immediately after surgery (T1) and six months after surgery (T2), to assess and record the translational and rotational changes of the condyle position. All the condyles showed lateral, forward, and downward rotation immediately after surgery. However, the condyles moved medially and upward after six months. The difference in the condylar position after six months and before surgery was less than 0.4 mm in all the rotational axes and spatial planes. Although the direction of translational changes was the same in both groups, the condyles in the group that had undergone LeFort I osteotomy showed greater bodily changes in terms of numeric values after six months compared to the preoperative state, but this difference was not statistically significant. Concerning the rotational changes of the condyle, the group that only underwent BSSO exhibited downward and medial rotation at six months after surgery, while the BSSO plus LeFort I group experienced upward and medial rotation. The magnitude of medial rotation was greater in the first group (BSSO only). Although both sides (with and without deviation) in both surgical groups exhibited lateral and downward movement and medial rotation of the condyles immediately after surgery, the magnitude of bodily movement on the non-deviated side was smaller in both groups (66% and 56% of the deviated side in BSSO alone and BSSO plus LeFort I groups, respectively). Eventually, no significant difference was noted in the magnitude of bodily movement between the two sides (with and without deviation from T0 to T2). One strength of this study, which made it unique, was the assessment of temporomandibular joint symptoms. Five condyles in the BSSO only group and one condyle in the BSSO plus LeFort I group showed symptoms, such as pain and clicking prior to surgery. After the surgery, however, only one condyle in the BSSO alone group was symptomatic. One asymptomatic condyle in the BSSO plus LeFort I group became symptomatic after the surgery. The authors could not draw any conclusions based on these findings. Despite their small sample size (n=18), they concluded that maxillary surgery combined with BSSO does not have a significant effect on changes in the position of the condyle (up to six months postoperatively), and these changes occur independently of the type of surgery.

### 3. Three-dimensional technology for the diagnosis, tre atment planning and prediction of surgical outcomes

Urib et al^21^ used 3D virtual treatment planning for two patients with facial asymmetry. One of the patients had class II, and the other had class III malocclusion, and both required bimaxillary surgery. The CBCT scan of the first patient was used to fabricate a composite model of the skull, and all the surgical movements were assessed using this model. The surgical splint was fabricated according to the 3D virtual treatment planning data by stone model surgery. For the second patient, the intermediate and final splints were designed using a completely digital model and fabricated using a 3D stereolithographic printer. In both patients, the superimposition of postoperative results on the initial condition using CBCT scans revealed no significant differences in the direction or magnitude of surgical movements, and the results were satisfactory. Evaluation of the accuracy of digitally fabricated surgical splints revealed that small differences between the prediction and final results could be due to orthodontic tooth movements after the surgical procedure. Therefore, they suggested comparisons at the level of osteotomy lines for further accuracy. According to Hsu et al,^52^ 3D planning enhances asymmetric surgical procedures by accurately and quantitatively determining the site of incision, osteotomy, and placement of screws and fixation plates in the three spatial planes.

Plooij et al^53^ recommended the integral fusion model as a method to collect information retrieved from the photographic records, CBCT scans, and dental models to enhance 3D surgical treatment planning, especially in patients with asymmetry.Janakiraman et al^20^ used this approach in a 23-year-old patient with unilateral condylar hyperplasia, who was a candidate for the SFA. In order to fabricate a fusion skull model, the CBCT scan data were combined with 3D photographs and digital dental models. Next, all the surgical movements of both jaws were designed by virtual surgical planning, and the absence of skeletal interferences during asymmetrical surgical movements was ensured. Condylectomy was designed by mirroring the normal condyle. Using the mirroring technique, the surgeon can compare both sides of the face quantitatively, and the normal side can serve as a guide for the surgeon to decide on the need for grafting, osteotomy, or other techniques to improve symmetry. Condylectomy was performed at the same time by orthognathic surgery, in contrast to Hsu et al,^52^ who claimed that in case of simultaneous conduction of orthognathic surgery and condylectomy, the relationship between the neuromuscular apparatus and the temporomandibular joint might be adversely affected.

In contrast to Uribe et al,^21^ Janakiraman et al^20^ performed soft tissue prediction using 3D software. Bianchi et al^54^ and Marchetti et al^55^ reported that the prediction of soft tissue changes three-dimensionally has an error rate of <2 mm in over 85% of the cases. In contrast, Terzic et al^56^ reported errors of >3 mm between the final soft tissue contour and predictions in almost 30% of the cases, which could have been due to the effect of variations in muscle tone, swelling, or head position in different individuals.^57^ Similarly, Janakiraman et al^20^ discussed that the accuracy of soft tissue prediction is a matter of question. In their study, not only the surgical treatment plan but also the orthodontic phase of the treatment process was virtually designed. The NiTi archwires were fabricated using SureSmile technology according to the predicted surgical results. They showed favorable stability at the 11-month follow-up and confirmed the results of previous studies, such as the study by De Riu et al,^58^ regarding 3D virtual planning.

There was also a valuable case report in this respect. Watanabe et al^22^ combined distraction osteogenesis and the SFA in five patients suffering from hemifacial microsomia. The entire surgical planning was carried out by 3D computer-assisted surgical simulation. All the patients were treated with the internal distractor and intermaxillary fixation, which remained for a minimum of four weeks after the completion of distraction. Since simultaneous maxillomandibular distraction has some shortcomings and can cause inaccuracy in proper positioning of the jaws, they suggested fixing the maxillary segment in a proper position by the SFA in the initial phase to prevent the above problems prior to the onset of distraction osteogenesis. In this method, the mandible is distracted towards the maxilla, which is located in an ideal position, using an internal device. This is guided by intermaxillary elastics. For correction of the asymmetrical cheek and paranasal contour, the maxillary jaw movements, and for improvement of oral symmetry, the rolling movements were used, which caused a significant improvement in the inter-commissural plane. The deviation decreased from 9° to 2.3°, with no unfavorable increase in the length of the midface.

As shown in Table 1, all the studies on the use of SFA in patients with asymmetry have been case reports or retrospective studies. Although these studies had some similarities in the treatment process, such as emphasis on the importance of 3D imaging, i.e., CBCT for assessment of asymmetry, or the critical role of rigid fixation for better stability, each of them discussed a different aspect of the problem, which highlights the lack of comprehensive studies on this topic. The largest sample size was 65 patients, which is not sufficient for reaching a definite conclusion. On the other hand, almost none of the reviewed studies had a long-term follow-up.

## Conclusion

Clinical trials following ethical principles and assessment of orthodontic and surgical factors that can affect the outcome of the SFA in asymmetric patients can provide a guideline for clinicians to help them through this process, from diagnosis to treatment planning and surgical procedure.

## Recommendations

We strongly suggest more detailed systematic reviews concerning SFA in different types of malocclusions and syndromes, its combination with DO approaches and ones with thorough discussions about the role of digital techniques in this field. By clarifying the advantages and indications of this method, significant progress can be made toward reduction of orthodontic iatrogenic effects and treatment duration.

## Author contributions

THN participated in the study concept and study design of this review. EG had a main role in the literature review and manuscript preparation. MAK contributed to the study design, literature review, and manuscript preparation. All authors have read and approved the final manuscript.

## Acknowledgment

We would like to acknowledge the outstanding contribution of Dr. Mojdeh Kalantar Motamedi for editing the English manuscript.

## Funding

Not applicable.

## Competing interests

The authors declare no competing interests with regard to the authorship and/or publication of this article.

## Ethical approval

Not applicable.
